# Encoding differences affect the number and precision of own-race versus other-race faces stored in visual working memory

**DOI:** 10.3758/s13414-017-1467-6

**Published:** 2018-01-17

**Authors:** Xiaomei Zhou, Catherine J. Mondloch, Stephen M. Emrich

**Affiliations:** Department of Psychology, Brock University, 1812 Sir Isaac Brock Way, St. Catharines, Ontario L2S 3A1 Canada

**Keywords:** Other-race effect, Visual working memory, Perceptual experience, Face recognition

## Abstract

Other-race faces are discriminated and recognized less accurately than own-race faces. Despite a wealth of research characterizing this other-race effect (ORE), little is known about the nature of the representations of own-race versus other-race faces. This is because traditional measures of this ORE provide a binary measure of discrimination or recognition (correct/incorrect), failing to capture potential variation in the quality of face representations. We applied a novel continuous-response paradigm to independently measure the number of own-race and other-race face representations stored in visual working memory (VWM) and the precision with which they are stored. Participants reported target own-race or other-race faces on a circular face space that smoothly varied along the dimension of identity. Using probabilistic mixture modeling, we found that following ample encoding time, the ORE is attributable to differences in the probability of a face being maintained in VWM. Reducing encoding time, a manipulation that is more sensitive to encoding limitations, caused a loss of precision or an increase in variability of VWM for other-race but not own-race faces. These results suggest that the ORE is driven by the inefficiency with which other-race faces are rapidly encoded in VWM and provide novel insights about how perceptual experience influences the representation of own-race and other-race faces in VWM.

Across a broad range of research paradigms investigating face recognition, there is a robust other-race effect (ORE), defined here as inferior performance when identifying faces of a different race than faces of the same race as the perceiver (see Bothwell, Brigham, & Malpass, 1989; Meissner & Brigham, 2001, for reviews).

In numerous studies examining the ORE, participants have been presented with own-race and other-race faces during a study phase and then asked to recognize those faces when they are intermixed with novel identities (the old/new face recognition task). A ubiquitous finding is that participants make more false alarms (incorrectly identifying an unseen face as familiar) and fewer hits (correctly identifying a previously seen face as familiar) for other-race compared to own-race faces, reflecting impairments in the encoding, storage and/or retrieval of other-race face representations from memory (Meissner & Brigham, 2001; Young, Hugenberg, Bernstein, & Sacco, 2012). A similar own-race advantage is found when learning is more extensive (e.g., Cambridge Face Memory Test, in which faces were learned from multiple angles; McKone et al., 2012), and when memory demands are minimized by asking participants to make same/different judgments for pairs of faces that differ only in feature shape or spacing (e.g., Hayward, Rhodes, & Schwaninger, 2008; Mondloch et al., 2010).

Although impaired memory for other-race relative to own-race faces is robust, traditional measures only provide a single binary measure of perceivers’ memory performance; each response is scored as either correct or incorrect. Such measures fail to capture potential variability in the quality of the representation, and so little is known about differences in the precision with which own-race and other-race faces are stored. The assumption that the representation of any given face stored in memory is a perfect representation is theoretically untenable and has recently been challenged by studies examining the precision with which basic visual features (colors, orientations) are stored in both visual working memory (VWM; Bays, Catalao, & Husain, 2009; Wilken & Ma, 2004; Zhang & Luck, 2008) and long-term memory (LTM; Brady, Konkle, Gill, Oliva, & Alvarez, 2013; also see Luck & Vogel, 2013, for a review).

A recent and more refined approach, the continuous response paradigm, provides a more sensitive index of the structure of memory (and perceptual) representations (Bays et al., 2009; Bays & Husain, 2008; Brady, Konkle, & Alvarez, 2011; Heyes, Zokaei, & Husain, 2016; Sarigiannidis, Crickmore, & Astle, 2016). In the continuous response paradigm, participants are asked to recall and report the remembered target, which is presented in an array of stimuli that vary along a continuous feature dimension (e.g., color, orientation). Response error is evaluated by calculating the angular deviation between the target item and the item reported by the participant. Probabilistic mixture modeling allows one to measure many sources of overall error (Bays et al., 2009; Bays & Husain, 2008; Brady et al., 2013), including (a) failure in encoding or retrieving the target item, leading to a random response (i.e., guessing); (b) noisiness of the stored representation, leading to decreased precision when the target is recalled; (c) trial-by-trial variability in the mean precision of those responses (i.e., how consistently the stored representation is recalled); and (d) representation of the target item being interrupted by a nontarget item, which leads to recalling the nontarget instead of the target (i.e., a swap error). Here, we used this methodological combination of continuous recall and mixture modeling to provide a more refined examination of the nature of own-race and other-race face representations, and the types of errors that lead to recognition impairments for other-race faces.

Although the continuous response paradigm has been widely used in studies examining VWM for basic features (e.g., hue, line orientation), its use with more complex stimuli is limited. Lorenc, Pratte, Angeloni, and Tong (2014) investigated the role of perceptual experience in encoding and storing face representations in VWM by contrasting VWM for upright versus inverted faces. It is widely established that inverted faces are discriminated and recognized less accurately than own-race faces; like the ORE, this inversion effect has been attributed to differential experience (Maurer, Le Grand, & Mondloch, 2002). Lorenc et al. reported a significant loss of precision for inverted faces relative to upright faces with no difference in the guess rate. The fidelity of representations in LTM is constrained by those in VWM (Brady et al., 2013). Thus, the difference in recognition performance between upright and inverted faces is partially attributable to the effect of visual experience on the fidelity of face representations encoded in VWM. Whether a similar difference in fidelity characterizes own-race compared to other-race faces remains unknown.

Here, we provide the first examination of the extent to which the ORE is attributable to a failure to encode and retrieve other-race faces from memory versus a loss of precision in their representations. To examine this question, we used a continuous response paradigm in which participants were asked to maintain own-race or other-race faces in VWM, and to report a target face on a unique circular face space that smoothly varied along the dimension of identity. The angular deviation between the target face and the face selected by the participant provides a more sensitive measure of face memory than can be obtained through traditional face recognition paradigms, as it captures continuous variability in face representations.

In two experiments, we examined the nature of the representations of own-race and other-race faces that are stored in VWM. In Experiment 1, we presented two faces on each trial, one of which was then cued for recall. By applying two different mixture models to the raw error, we differentiated potential sources of error that contribute to the ORE: random guesses, swap errors, and lack of precision and/or trial-by-trial variability in precision for a remembered face. In Experiment 2, we presented only one face but varied presentation time. Applying mixture modeling here allowed us to examine whether reducing presentation time especially impaired VWM for other-race faces.

## Experiment 1: Storing two faces with ample encoding time

### Method

#### Participants

Fifteen Caucasian adults (one male, ages 19–30 years, *SE* = 0.68) from Brock University participated in the study and were included in the final analysis, a sample size comparable to that in other studies using the continuous response paradigm (Brady et al., 2013; Lorenc et al., 2014). All participants reported minimal contact with other-race identities and verbally confirmed normal or corrected-to-normal vision. An additional seven participants were excluded from the final analysis because they reported extensive contact with Asian identities (*n* = 1) or had extremely poor performance (i.e., guess rate exceeded 2.5 standard deviations of the mean; *n* = 6). All participants provided written informed consent and received either research credit or a small honorarium for their participation. This study received clearance from the Research Ethics Board at Brock University.

#### Stimuli

Four Caucasian and four East Asian faces were acquired from the Let’s Face It database at Brock University. All faces were female, physically similar, displayed in full-front view and unfamiliar to the participants. Each identity was paired with each of the other same-race identities to create six pairings. We then used a linear morphing procedure to create 19 morphed faces for each pairing by blending the two faces in 5% steps (e.g., 95/5, 90/10, . . . , 5/95). Nineteen morphs across six face pairs for each of the two race categories resulted in a total of 236 faces (228 morphs; eight originals) that were used in the experiment.

A unique circular face space comprised of Caucasian or East Asian faces, analogous to a color wheel, was created on each trial by randomly placing the four original (anchor) faces with equal distances between them. Based on their relative location, morphed faces were then placed among the anchor faces such that identity varied continuously around the wheel. Because all faces used to create the face wheel were wholly unfamiliar to our participants, no face on the wheel had special status (i.e., categorical perception was precluded). Thus, in the 360° circular face space, 80 faces (four anchors; 76 morphs) were evenly distributed, making the difference between any two neighboring faces equivalent to 4.5°. All faces were standardized at 395 × 510 pixels and were presented on a 19-inch computer monitor with the viewing distance approximately 60 cm. Stimuli were presented, and participants’ response were collected using PsychoPy1.8 (Peirce, 2007, 2009).

#### Procedure

Each participant completed a 1-hour session, comprising eight practice trials (four/race) followed by 240 test trials. The race of face was blocked such that half of the participants were presented with Caucasian faces first and the other half with East Asian faces first.

**Fig. 1:**
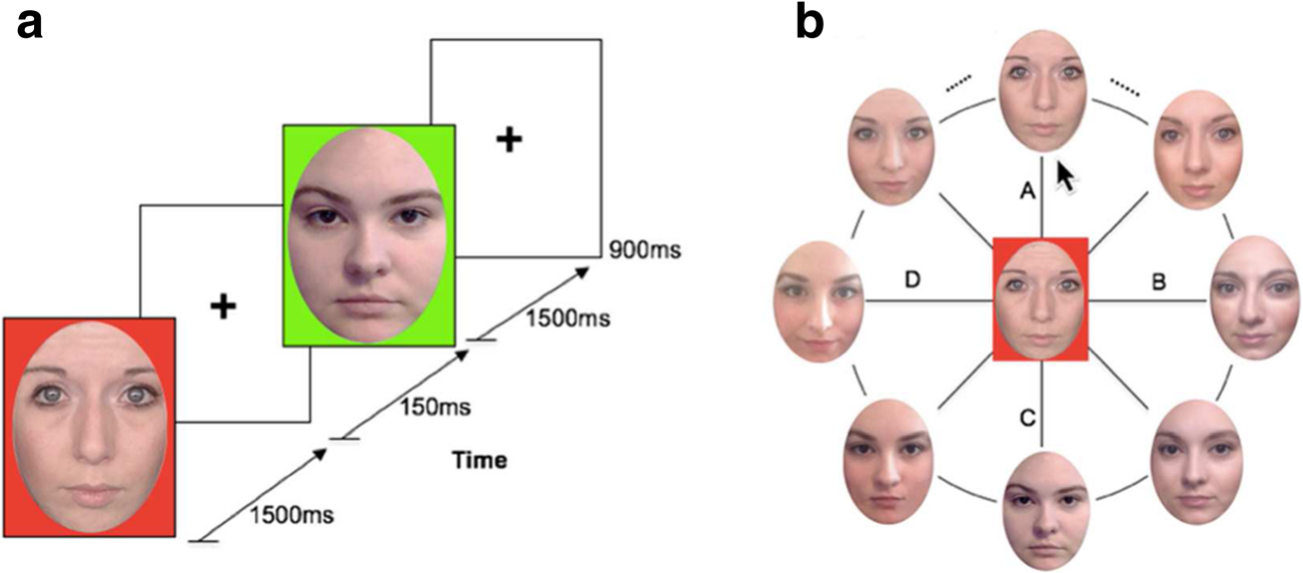
Continuous response task used in the first experiment. **a** Two study faces, each of which was paired with a cue color **b** Response phase. Target face was cued by a color (e.g., red), and when participants moved the mouse along the face wheel, the face in the center changed simultaneously to indicate the face that they were reporting. *Note.* Permissions preclude showing faces used in the actual study; faces in here are for demonstration only. (Color figure online)

Each trial began with a sequential presentation of two faces (e.g., 90%A–10%B; 55%C–45%D) that were chosen randomly from the face space (could be anchor or morphed faces), followed by a delay period of 900 ms, and then a face wheel (see Fig. 1). The two faces were cued by different colors (red or green) and were presented sequentially for 1,500 ms each, with a 150-ms interstimulus interval. A 1,500 ms presentation time ensures full encoding of each face in VWM (Lorenc et al., 2014). One of the two faces was randomly assigned as the target face and the other as the nontarget face. Participants were unaware of which face was the target and were instructed to memorize both of them. After the 900-ms delay, a red or green rectangle appeared in the center of the screen indicating which face was the target. Eight randomly chosen and equidistant faces from the face wheel were presented around the central target item at equal intervals. Participants were instructed to locate the target face by using a computer mouse to select a point on the face wheel. While they moved the mouse along the face wheel, the face in the center changed simultaneously to indicate the face they were selecting. Like the composition of the face wheel, both the color (red/green) and the position (first/second) of the target were randomized across trials. Participants proceeded at their own pace and were asked to be as accurate as possible in their decision.

### Data analysis

#### Overall response error

Response error was calculated for each trial as the angular deviation (in degrees; −180° to 180°) between the correct orientation of the target face and the orientation of the face reported by the participant. To obtain a generic measure of the overall precision of response, we calculated the reciprocals of the standard deviation (1/*SD*) of response error across trials separately for own-race and other-race faces.

To further identity the sources of increased response error for other-race faces, we fit the raw error using two models: a variable-precision model, in which precision of face representations varies across items and trials (Fougnie, Suchow, & Alvarez, 2012; van den Berg, Shin, Chou, George, & Ma, 2014) and an equal-precision model, in which each face representation is assumed to have equal precision (Bays & Husain, 2008; Zhang & Luck, 2008). The general method for both model types involves finding the maximum likelihood of a mixture of distributions, which are fit to the raw error.

#### Variable-precision model

In the case of the variable-precision model, the precision of responses is assumed to vary according to a higher order, truncated normal distribution (Fougnie et al., 2012). The model therefore takes the following form:

$$ p\left(\widehat{\theta}\right)=\left(1-\gamma \right)\psi \left(\widehat{\theta}-\theta \right)+\gamma \frac{1}{2\pi } $$

where *γ* represents the proportion of trials on which the participant is randomly guessing (i.e., a flat distribution). The error term on the remaining trials is defined as the difference between the target face (*θ*) and the face selected by the participant ($\widehat{\theta}$); these responses fall under a wrapped Student’s *t* distribution (*ψ*). The model, therefore, returns three parameters of interest: the proportion of trials on which the participant is assumed to be guessing (*γ*), the mean standard deviation of responses on remaining trials (trials on which they did report the target; inverse of precision), and the standard deviation of response error on these remaining trials (reflecting intertrial variability in precision). A larger standard deviation of response error indicates more variability in the quality of the face representation stored across items and trials.

#### Equal-precision model

We also fit an equal-precision model to each participant data set for own-race and other-race faces. We used the three-component model (Bays et al., 2009; Bayes, Gorgoraptis, Wee, Marshall, & Husain, 2011), described by the following equation:

$$ p\left(\widehat{\theta}\right)=\alpha {\phi}_{\kappa}\left(\widehat{\theta}-\theta \right)+\beta \frac{1}{m}\ \sum \limits_i^m{\phi}_{\kappa}\left(\widehat{\theta}-{\varphi}_i\right)+\gamma \frac{1}{2\pi } $$

where *α*, *β*, and *γ* represent the probability of reporting the correct target face, the probability of mistakenly reporting the nontarget face, and the probability of responding randomly, respectively. Here, *α* + *β* + *γ* = 1. In addition, *θ* represents the correct location of the target face, and $\widehat{\theta}$ represents the location of the face reported by the participant. The von Mises (circular normal) distribution is *ϕ*_*κ*_,with the mean zero and the concentration parameter *κ*. Greater *κ* indicates a more concentrated von Mises distribution. The number of nontarget faces is *m*, in this case, *m* = 1, and {*φ*_1_, *φ*_2_, …*φ*_*m*_} are the locations of the m nontarget faces. Thus, according to this model, the overall response distribution comprises a mixture of three components (Bays et al., 2009): (1) the proportion of trials on which the participant is assumed to be guessing; (2) target (correct) responses, from a von Mises distribution centered on the target face, indicating the probability that perceivers correctly remembered the target face; and (3) nontarget responses, drawn from the same von Mises distribution but centered on the nontarget face (i.e., the distractor face), indicating the probability of a swap error.

The proportion of correct responses can also be transformed into an estimate of the number of successfully maintained faces by multiplying the probability of correct responses by the set size (e.g., *n* = 2 in Experiment 1) for both own-race and other-race faces.

For all model fits, maximum likelihood estimates of the mixture parameters for each participant and face race were obtained using an expectation-maximization algorithm implemented with the MemToolBox 1.0 (Myung, 2003; Suchow, Brady, Fougnie, & Alvarez, 2013).

### Results

#### Overall response error

**Fig. 2:**
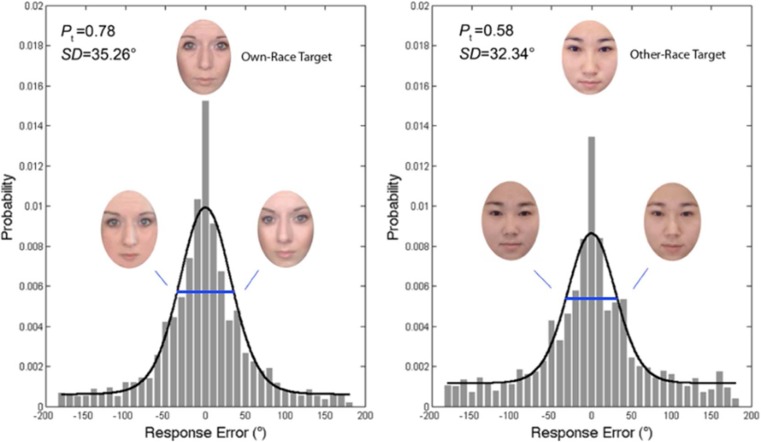
Distribution of response errors for own-race (left) and other-race (right) faces. Histogram displays the proportion of binned response errors relative to the target face. Black lines display the three-component mixture model, fit to the raw error. Blue lines indicate the width of the von Mises (circular normal) distribution at 1 standard deviation and are flanked by corresponding own-race and other-race identities (±1 *SD* of error). P_t_ indicates proportion of correctly reported targets, and *SD* indicates 1 standard deviation of the circular error for these responses. The additional peakiness surrounding the zero-target value is better accounted for by the variable precision model (not pictured). *Note.* Permissions preclude showing faces used in the actual study; faces here are for demonstration only. (Color figure online)

The distribution of errors for own-race and other-race faces is shown in Fig. 2. A paired-samples *t* test revealed a significant main effect of face race, *t*(14) = 3.69, *p =* .002, Cohen’s *d* = 0.95; overall, participants had smaller response errors for own-race faces (*M*_*SD*_ = 56.61^o^) than for other-race faces (*M*_*SD*_ = 69.66^o^).

#### Variable-precision model

In order to examine the trial-to-trial variability in precision, as well as the proportion of trials in which participants guessed, a variable-precision model was fit to the raw error (see Table 1 for parameter means). Paired-samples *t* tests revealed a significantly higher guess rate for other-race faces than for own-race faces, *t*(14) = 3.57, *p =* .003, Cohen’s *d* = 0.92, with no difference in precision of VWM for own-race versus other-race faces, *t*(14) = 0.67, *p* = .514, Cohen’s *d* = 0.17, and no difference in variability in the precision of VWM for own-race versus other-race faces, *t*(14) = 0.634, *p* = .537, Cohen’s *d* = 0.16.

**Table 1:** Mean (and *SD*) of variable precision model parameters (Experiment 1)

Parameter	Face condition	Mean (*SD*)
Guess rate	Own	0.160 (0.150)
	Other	0.336 (0.220)
Mean *SD* (degrees)	Own	42.947 (24.323)
	Other	38.179 (27.729)
*SD* variance (degrees)	Own	23.207 (29.106)
	Other	30.773 (37.023)

#### Equal-precision model

This pattern was confirmed using the equal-precision model. The result of the model fit is plotted in Fig. 2. Paired-samples *t* tests revealed a lower correct response rate for other-race faces (*M* = .58) than for own-race faces (*M* = .78), *t*(14) = 3.57, *p =* .003, Cohen’s *d* = 0.95. The significant difference in the proportion of correct responses was attributable to a significant difference in guess rate (*M* = .24 vs .03 for other-race vs. own-race faces), *t*(14) = 3.36, *p =* .005, Cohen’s *d* = 0.88, with no difference in swap errors (*M* = .18 vs .19 for other-race vs. own-race faces), *t*(14) = 0.17, *p =* .865, Cohen’s *d* = 0.04. The change in guess rate reflects a diminished number of stored faces for other-race (*k* = 1.16) relative to own-race (*k* = 1.56) faces. Notably, we did not detect any difference between the precision of VWM for own-race and other-race faces, *t*(14) = 0.74, *p =* .472, Cohen’s *d* = 0.19, as indicated by comparable standard deviations of von Mises distributions for own-race faces (35.26^o^) and other-race faces (32.34^o^).

### Discussion

When holding two potential target faces in VWM and given ample encoding time, participants made significantly larger errors in their recall of other-race compared to own-race faces, as indicated by the greater angular deviations (*SD*) between the target face and the face that was reported by the participant. Results of both variable-precision and equal-precision modeling further informed us that the increase in overall errors for other-race faces was attributable to an increased guess rate but not to reduced precision or an increase in swap errors. Under these task conditions, differences in performance between own-race and other-race faces can be attributed to impairments in the encoding, consolidation, and/or retrieval of other-race face representations, rather than a change in either the precision with which remembered faces are stored or an increase in identity confusion.

## Experiment 2: Storing one face with limited encoding time

In Experiment 1, participants were given ample time (1,500 ms) to encode each of two faces; one face was then cued for recall. This protocol is maximally sensitive to storage limitations (Bays et al., 2011) and also enabled us to examine the contribution of interference by other faces to the ORE. Encoding limitations are best captured by very brief presentations (Bays et al., 2011). To examine whether any observed differences in Experiment 1 were attributable to differences in encoding, in Experiment 2 we examined whether reducing presentation time (from 1,500 to 200 ms) especially impairs the probability and/or precision of correct responses for other-race faces. To isolate limitations in encoding, we further reduced the set size to one, thus working well below the capacity of VWM observed in Experiment 1.

### Method

#### Participants

Twenty Caucasian adults (four males, ages 18–25 years, *SE* = 0.45) from Brock University participated in the study.

#### Stimuli and procedure

The stimuli and procedure were identical to Experiment 1, with three exceptions: There were 420 test trials, only one face was presented on each test trial, and the presentation time of the target face varied across trials. On half of the trials faces were presented for 200 ms, and on the other half for 1,500 ms (as in Experiment 1).

#### Data analysis

All analyses were performed identically to those in Experiment 1, with one exception: Given the absence of a nontarget face in Experiment 2, a two-component mixture model proposed by Zhang and Luck (2008) was used. The components in this model are comparable to those in the three-component model, but swap errors are removed. The components are described by the following equation (here,*α* + *γ* = 1):

$$ p\left(\widehat{\theta}\right)=\alpha {\phi}_{\kappa}\left(\widehat{\theta}-\theta \right)+\gamma \frac{1}{2\pi } $$

#### Results

ᅟ

#### Overall response error

**Fig. 3:**
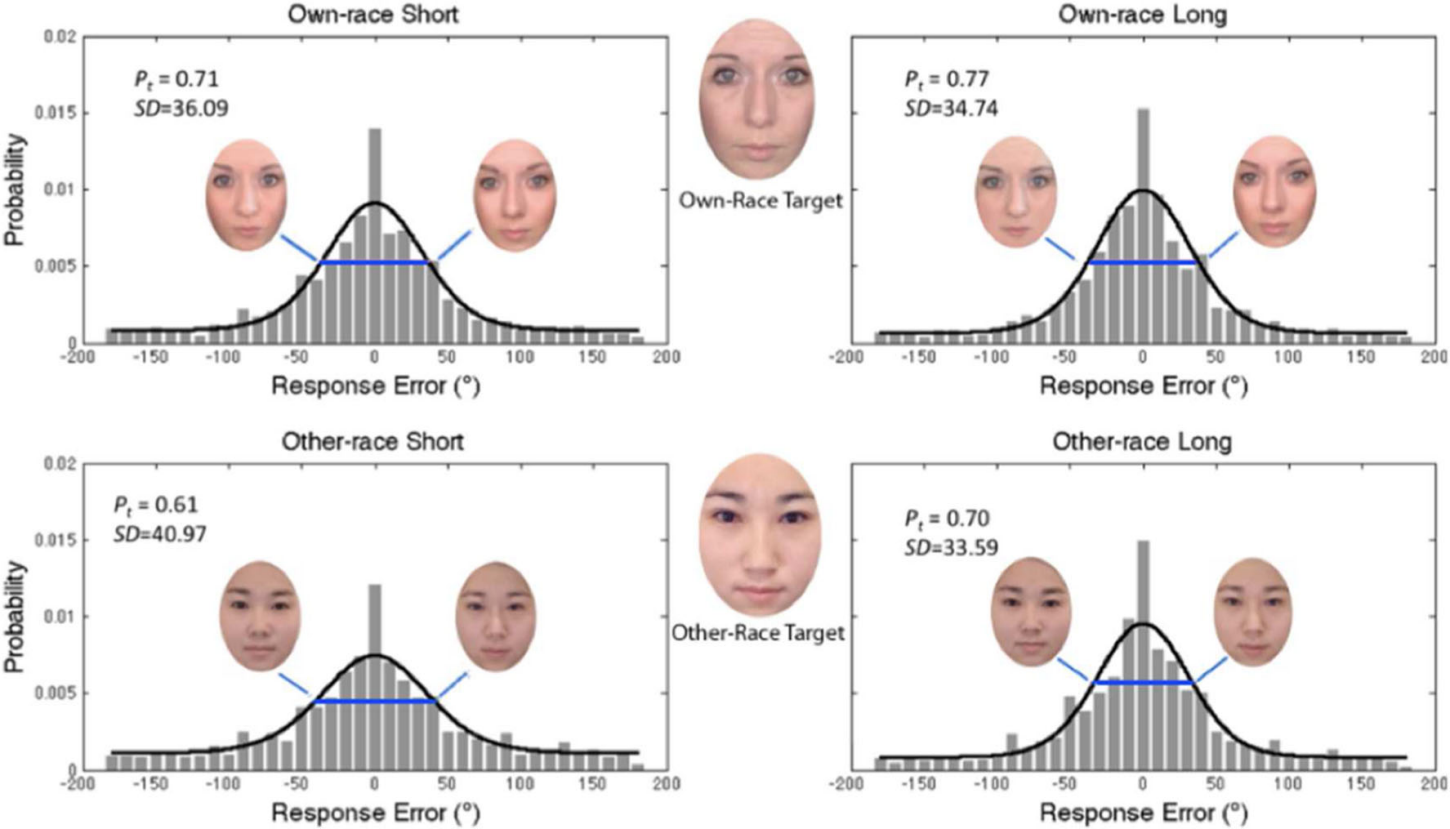
Distribution of response error for own-race (top) and other-race (bottom) faces when the faces were presented for 200 ms (left) and 1,500 ms (right). Histograms display proportion of binned responses relative to the target face. Black lines display the two-component mixture model, fit to the raw response error. Red solid lines indicate the width of the von Mises (circular normal) distribution at 1 standard deviation and are flanked by corresponding own-race and other-race identities (±1 *SD* of error). *P*_*t*_ indicates the proportion of correctly reported targets, and *SD* indicates 1 standard deviation of the circular error for these responses. The additional peakiness surrounding the zero-target value is better accounted for by the variable precision model (not pictured). *Note.* Permissions preclude showing the faces used in the actual study; faces here are for demonstration only. (Color figure online)

The distribution of errors from Experiment 2 is displayed in Fig. 3. A 2 (face race: own-race vs. other-race faces) × 2 (presentation time: 200 ms vs. 1,500 ms) repeated-measures ANOVA revealed significant main effects of face race, *F*(1, 19) = 7.68, *p* = .012, η_p_^2^ = .29, and presentation time, *F*(1, 19) = 40.26, *p* < .001, η_p_^2^ = .68. Overall response error was lower for own-race faces (*M*_*SD*_ = 59.57^o^) than for other-race faces (*M*_*SD*_ = 65.89^o^) and when faces were presented for longer time (*M*_*SD*_ = 58.69^o^) than when faces were presented for shorter time (*M*_*SD*_ = 66.77^o^). The Face Race × Presentation Time interaction did not reach significance, *F*(1, 19) = 1.85, *p* = .190, η_p_^2^ = .09. Thus, independent of the length of encoding time, participants demonstrated greater error in their recall of other-race compared to own-race faces, consistent with the ORE.

#### Variable-precision model

A 2 (face race: own-race vs. other-race faces) × 2 (presentation time: short vs. long) repeated-measures ANOVA, with guess rate as the dependent variable, revealed a significant main effect of presentation time, *F*(1, 19) = 10.72, *p* = .004, η_p_^2^ = 0.36, but no main effect of face race, *F*(1, 19) = 0.837, *p* = .372, ηp^2^ = 0.04, and no Face Race × Presentation Time interaction, *F*(1, 19) = 0.18, *p* = .674, η_p_^2^ = 0.01. Participants had a higher guess rate for the shorter presentation time than for the longer presentation, but reducing presentation time did not particularly impair the probability of other-race faces being recalled from VWM (see Table 2 for parameter means).

**Table 2:** Mean (and *SD*) of variable precision model parameters (Experiment 2)

Parameter	Encoding duration	Face condition	Mean (*SD*)
Guess rate	Short	Own	0.248 (0.228)
		Other	0.273 (0.189)
	Long	Own	0.155 (0.206)
		Other	0.195 (0.184)
Mean *SD* (degrees)	Short	Own	38.792 (9.992)
		Other	60.703 (25.718)
	Long	Own	41.072 (17.413)
		Other	40.008 (15.544)
*SD* variance (degrees)	Short	Own	10.788 (10.582)
		Other	35.393 (30.432)
	Long	Own	19.007 (16.934)
		Other	25.674 (22.080)

A 2 (face race) × 2 (presentation time) repeated-measures ANOVA, with mean precision of responses as the dependent variable revealed, significant main effects of face race, *F*(1, 19) = 5.29, *p* = .033, η_p_^2^ = 0.22, and presentation time, *F*(1, 19) = 6.70, *p* = .018, ηp^2^ = 0.26. The fidelity of own-race faces stored in VWM was greater than that of other-race faces and, overall, the fidelity of faces was greater for longer than for shorter presentation times. Notably, we found a significant interaction between face race and presentation time, *F*(1, 19) = 12.06, *p* = .003, ηp^2^ = 0.39. For own-race faces, the precision of VWM was comparable for short (*M* = 38.8°) and long (*M* = 41.1°) presentation time, *t*(19) = 0.557, *p* = .584, Cohen’s *d* = 0.125; however, reducing presentation time particularly impaired the fidelity of representations of other-race faces stored in VWM (*M =* 60.7° and 40.0°*,* respectively), *t*(19) = 3.751, *p* = .001, Cohen’s *d* = .839.

A 2 (face race) × 2 (presentation time) ANOVA, with variability in precision as the dependent variable, revealed a main effect of face race, *F*(1, 19) = 8.52, *p* = .009, η_p_^2^ = 0.31; variability in precision was higher for other-race than for own-race faces. Although the main effect of presentation time was not significant, *F*(1, 19) = 0.02, *p* = .888, ηp^2^ = 0.001, there was a significant interaction, *F*(1, 19) = 4.70, *p* = .043, ηp^2^ = 0.20. Although the variability in other-race faces was not significantly greater for short compared to long presentation times, *t*(19) = 1.113, *p* = .279, *d* = 0.249, variability was significantly greater for other race faces compared to own race faces when encoding time was reduced, *t*(19) = 3.369, *p* = .003, *d* = .753, but not when given ample encoding time, *t*(19) = 1.078, *p* = .295, *d* = .241.Thus, consistent with the effects on mean standard deviation, reducing encoding time selectively impaired the recall of other-race faces.

#### Equal-precision model

Similar results were obtained using an equal-precision model. A 2 (face race: own-race vs. other-race faces) × 2 (presentation time: short vs. long) repeated-measures ANOVA, with proportion of correct responses as the dependent variable, revealed significant main effects of face race, *F*(1, 19) = 7.87, *p* = .011, ηp^2^ = .29, and presentation time, *F*(1, 19) = 17.90, *p* < .001, ηp^2^ = .49. As shown in Fig. 3, participants made significantly fewer correct responses for other-race faces (*M* = .66) than for own-race faces (*M* = .74) and for the shorter presentation time (*M* = .66) than for the longer presentation time (*M* = .74). Consequently, the number of recalled faces was lower for other-race (*k* = .66) than for own-race (*k* = .74) faces and for shorter presentation time (*k* = .66) than for longer presentation time (*k* = .74). Notably, the Face Race × Presentation Time interaction did not approach significance, *F*(1, 19) = 1.27, *p* = .274, ηp^2^ = .06, indicating that reducing presentation time did not especially impair the probability of an other-race face being recalled.

Consistent with the variable-precision model, results of the equal-precision model suggested that the precision of VWM (1/*SD* of the von Mises distribution) was greater for the longer presentation time (*M*_*SD*_ = 34.17^o^) than the shorter presentation time (*M*_*SD*_ = 38.53^o^), as revealed by the significant main effect of presentation time, *F*(1, 19) = 7.51, *p* = .013, ηp^2^ = .28. The main effect of face race was not significant, *F*(1, 19) = .67, *p* = .424, ηp^2^ =.03, but the interaction between face race and presentation time approached significance, *F*(1, 19) = 3.31, *p* =.085, ηp^2^ =.15. Based on a priori hypotheses and the results of the variable precision model, we conducted paired-samples *t* tests; these confirmed that reducing presentation time significantly reduced precision for other-race faces (*M*_*SD*_ = 40.97° vs. 33.59° for 200 vs. 1,500 ms), *t*(19) = 2.82, *p =* .011, Cohen’s *d* = 0.63. In contrast, precision was comparable for shorter (*M*_*SD*_ = 36.09°) and longer presentation times (*M*_*SD*_ = 34.74°) for own-race faces, *t*(14) = 0.70, *p =* .494, Cohen’s *d* = 0.16.

### Discussion

Overall, participants’ precision of recall was impaired when encoding time was reduced to 200 ms and when encoding other-race compared to own-race faces. Under conditions that were maximally sensitive to encoding limitations, mixture modeling revealed that the increase in response error was driven by a decrease in the probability of a correct response for both own-race and other-race faces. For other-race faces only, we also observed a reduction in the fidelity of representation and in increase in the trial-by-trial variability in fidelity when encoding time were reduced.

## General discussion

In summary, using a novel continuous response paradigm, we provided the first evidence that the ORE is attributable to increased error in the representation of other-race faces in VWM. We then used mixture modeling to examine how different sources of error contribute to the ORE: a failure to encode and retrieve other-race face representations (guess rate), increased interruption from nontarget faces (identity confusion), reduced precision for other-race faces, and/or increased variability in the precision with which faces are represented. Based on this analysis, we revealed three novel findings. First, following ample exposure to multiple own-race and other-race faces, the ORE was evident in an increased guess rate but not in reduced precision or an increase in identity confusion. Second, limiting encoding time impaired precision for a single other-race but not own-race face. Third, limiting encoding time increased variability in the precision with which other-race faces were represented in VWM. Collectively, these results suggest that the ORE is caused by a failure to rapidly consolidate other-race faces into coherent and stable representations in VWM.

Our findings build on two previous studies showing that perceptual experience affects how faces are stored in VWM (Humphreys, Hodsoll, & Campbell, 2005; Lorenc et al., 2014). To the best of our knowledge, the only previous study to explicitly contrast VWM for own-race and other-race faces used the change blindness paradigm (Humphreys et al., 2005). These authors reported faster change detection for own-race than for other-race faces, but the change blindness paradigm precludes examining the separate contributions of a failure to encode and retrieve other-race faces versus reduced fidelity in their representation.

Lorenc et al. (2014) used the continuous response paradigm to compare VWM for upright and inverted faces (two face categories with which adults have differential experience). Precision, but not capacity, of VWM was greater for upright faces. Here, for the first time, we applied the continuous response paradigm to examine the ORE. Like Lorenc et al., we found that perceptual experience influences the precision of VWM for faces; reducing presentation time to 200 ms impaired precision for other-race, but not for own-race, faces. Unlike Lorenc et al., we also found that experience influences the number of faces that can be maintained in VWM. These differential patterns might reflect a difference between the two studies in the dimensions along which faces continuously varied rather than differential effects for orientation versus face race: Whereas the faces in Lorenc et al.’s study varied in both age and sex, ours differed only in identity. Encoding and maintaining sex and age in VWM might be easier than encoding and maintaining identity, as suggested by both fewer correct responses and greater variability of face representations reported for participants in our study. Nonetheless all of these studies provide strong evidence that VWM for faces is impacted by experience.

The inefficiency with which other-race faces are rapidly encoded and consolidated into stable representations is consistent with a large body of electrophysiological studies examining the neural mechanisms of the ORE. These studies reported smaller amplitudes of N170 and P200 for other-race than own-race faces (Ito & Urland, 2005; Senholzi & Ito, 2012; Vizioli, Foreman, Rousselet, & Caldara, 2009; Vizioli, Rousselet, Foreman, & Caldara, 2009; but see Balas & Nelson, 2010; Herrmann et al., 2007; Stahl, Wiese, & Schweinberger, 2008)—ERP components that peak over temporo-occipital brain regions about 170 ms and 200 ms after stimulus onset. N170 and P200 are thought to reflect structural encoding of faces (i.e., processing physiognomic information to form a sensory representation) and configural processing (i.e., integrating facial features into a whole). These electrophysiological studies suggest reduced efficiency in structural encoding and configural processing for other-race faces, consistent with behavioral evidence (see Michel, Rossion, Han, Chung, & Caldara, 2006; Mondloch et al., 2010; Rhodes, Hayward, & Winkler, 2006; Tanaka, Kiefer, & Bukach, 2004).

The other-race effect was revealed in two different measures across Experiments 1 and 2, a pattern we attribute to details in task parameters. In Experiment 1, we used task parameters that maximize sensitivity to storage limitations (Bays et al., 2011); we provided ample encoding time (based on Lorenc et al., 2014) and presented two faces on each trial. Under these task conditions, we found differences in the probability of a face being recalled (correct response rate), with no difference in precision or in swap errors (i.e., confusing one identify for another). In other words, when participants had ample time to encode multiple faces, they reported fewer other-race faces, an effect that suggests fewer other race faces were stored in VWM—although it is not clear whether this effect was driven by differences in encoding, consolidation, or storage capacity differences. Experiment 2 was designed to be especially sensitive to any differences with which own-race versus other-race faces are *encoded*. We presented only one face per trial (working well below the capacity of VWM observed in Experiment 1, thus isolating the effect of face race on encoding limitations) and included very brief presentations (Bays et al., 2011). Under these task conditions, we found reduced precision for other-race faces but not for own-race faces when encoding time was limited. Thus, whether the other-race effect is reflected in the probability or precision of correct responses depends on task parameters. When multiple faces were presented, participants were able to store fewer representations of other-race faces; if only a single face was presented, however, participants were not limited by storage differences but instead were affected by manipulations that targeted encoding limitations, revealing differences in the fidelity of the encoded representations. This finding is consistent with previous findings that familiar exemplars of a perceptual category are encoded more quickly than unfamiliar exemplars (Xie & Zhang, 2017b).

The differences in encoding of representations of own-race and other-race faces from VWM likely reflect asymmetric perceptual experience faces from these two categories. Complex objects (e.g., Chinese characters, random polygons) place greater demands on VWM than do simple objects, leading to a reduced VWM capacity (Alvarez & Cavanagh, 2004; also see Brady et al., 2011, for a review). Although own-race and other-race faces do not differ in stimulus complexity, as evident in the ORE being independent of race of face and race of participants (e.g., Ng & Lindsay, 1994; Sporer, 2001), limited perceptual experience with other-race faces likely increases the demands on VWM, one consequence of which appears to be a reduction in the precision with which other-race faces are stored in VWM.

The observed differences in VWM for own-race versus other-race faces are consistent with other evidence of an own-race advantage. Indeed, differences in VWM likely originate in, and contribute to, differences in perception and mental representation. According to Valentine’s influential norm-coding model (Valentine, 1991), faces are represented in a multidimensional face space and are encoded with reference to their deviation from a face prototype/norm that represents the average of all faces previously encountered. Individual differences in norm-based coding correlate with individual differences in recognition accuracy (Dennett, McKone, Edwards, & Susilo, 2012; Rhodes, Jeffery, Taylor, Hayward, & Ewing, 2014), suggesting that representing individual faces relative to a prototype enhances sensitivity to subtle differences among them. One explanation for the ORE is that other-race faces are more densely clustered in psychological space (Byatt & Rhodes, 1998; Papesh & Goldinger, 2010; Zhou, Short, Chan, & Mondloch, 2016), making them harder to discriminate (e.g., Mondloch et al., 2010). Impaired VWM for other-race faces likely contributes to the mental representation of other-race faces being less refined; this, in turn, likely impacts how other-race faces are represented in VWM. Likewise, the representation of other-race faces in VWM likely impacts, and is impacted by, the accuracy with which faces are stored in LTM. The number of stimuli maintained in VWM is influenced by familiarity within LTM (Xie & Zhang, 2017a), and the fidelity of LTM representations is constrained by those encoded and maintained in VWM (Brady et al., 2013).

### Limitations and future directions

Of necessity we presented identical images of unfamiliar identities at study and test. The function of face perception in daily life, however, is to recognize familiar identities despite within-person variability in appearance (e.g., in lighting, hairstyle, expression, viewpoint; Burton, 2013). Impairments in VWM for other-race faces might contribute to increased errors in recognizing that two different images of an unfamiliar other-race face belong to the same identity (Laurence, Zhou, & Mondloch, 2016) and likely impact processes by which a newly encountered face becomes familiar (e.g., ensemble encoding—the rapid and automatic formation of an average; Kramer, Ritchie, & Burton, 2015). Directly examining how VWM impacts face learning would be a fruitful line of research.

Ideally, studies investigating the ORE in face perception include a complete cross-over design (i.e., test both Caucasian and Eastern Asian participants) to avoid any possibility of a stimulus effect. We controlled for stimulus effects by using the same faces under two different task conditions; nonetheless, future studies should incorporate testing East Asian participants*.*

Moreover, note that the maximum number of faces to be remembered in VWM in the current study was two; future studies should examine how the precision of VWM for own-race and other-race faces changes across a larger range of set sizes, and examine whether VWM for more complicated visual stimuli such as own-race and other-race faces is limited by a discrete or continuous resource (van den Berg, Awh, & Ma, 2014). Future studies might provide a more refined measure of the precision of VWM for own-race versus other-race faces by reducing the physical difference between adjacent faces in the wheel (i.e., by using 1-degree steps and adding more faces to the wheel)*.*

Finally, it is important to note that although we observed similar effects employing two different mixture models—namely, the variable-precision and equal-precision models—it remains possible that other models might better explain memory for faces. Indeed, in the VWM field, numerous factors have been examined in an attempt to best explain memory performance, including the presence or absence of capacity limits, variability, nontarget errors, categorical perception, and interference (Hardman, Vergauwe, & Ricker, 2017; Oberauer & Lin, 2017; van den Berg et al., 2014). Moreover, although variable precision models have typically best accounted VWM performance (Fougnie et al., 2012; van den Berg et al., 2014; van den Berg et al., 2012), performance can still be affected by additional factors, such as the allocation of attention during encoding (Emrich, Lockhart, & Al-Aidroos, 2017). It is also important to note that all faces used to create the wheel were unfamiliar to our observers, precluding categorical encoding of identity. Future studies using familiar faces (for which categorical encoding is inevitable) will benefit from a recent model proposed by Hardman et al. (2017) that includes a categorical component in the model. While future studies may examine different models, the fact remains that employing the methods used here and in Lorenc et al. (2014) provide a much finer tool for examining the nature of face representations in memory and perception than do traditional measures, such as the old/new recognition task.
